# Evaluation of a laying-hen tracking algorithm based on a hybrid support vector machine

**DOI:** 10.1186/s40104-016-0119-3

**Published:** 2016-10-12

**Authors:** Cheng Wang, Hongqian Chen, Xuebin Zhang, Chaoying Meng

**Affiliations:** 1 College of Information and Electrical Engineering, China Agricultural University, Beijing, 100083 China; 2 Network Center, China Agricultural University, Beijing, 100083 China; 3 College of Water Resources and Civil Engineering, China Agricultural University, Beijing, 100083 China

**Keywords:** Computer vision, Laying hens, Locomotion tracking, Support vector machine

## Abstract

**Background:**

Behavior is an important indicator reflecting the welfare of animals. Manual analysis of video is the most commonly used method to study animal behavior. However, this approach is tedious and depends on a subjective judgment of the analysts. There is an urgent need for automatic identification of individual animals and automatic tracking is a fundamental part of the solution to this problem.

**Results:**

In this study, an algorithm based on a Hybrid Support Vector Machine (HSVM) was developed for the automated tracking of individual laying hens in a layer group. More than 500 h of video was conducted with laying hens raised under a floor system by using an experimental platform. The experimental results demonstrated that the HSVM tracker outperformed the Frag (fragment-based tracking method), the TLD (Tracking-Learning-Detection), the PLS (object tracking via partial least squares analysis), the MeanShift Algorithm, and the Particle Filter Algorithm based on their overlap rate and the average overlap rate.

**Conclusions:**

The experimental results indicate that the HSVM tracker achieved better robustness and state-of-the-art performance in its ability to track individual laying hens than the other algorithms tested. It has potential for use in monitoring animal behavior under practical rearing conditions.

## Background

The behavior of animals is an important indicator of their welfare [1, 2]. Animal behavior is typically monitored through manual observation which requires substantial manpower and cannot always guarantee accuracy [3]. The demand for methods to automatically monitor animal behavior and track their movement has recently been increasing thereby promoting the initiation of related research [4].

Previous studies of animal behavior have focused on two main objectives, namely the identification of specific behavior and the tracking of animal movement. With respect to behavioral identification, the appearance of animals varies widely depending on their location which renders image processing and interpretation very difficult [5]. Some researchers have identified the behavior of animal groups through visual techniques such as monitoring the weight distribution in poultry flocks [6, 7], the spatial distribution of pigs [8, 9], the distribution of broilers [10], and the trajectory of a flock of poultry [11].

Monitoring the behavior of a particular animal in a group requires information obtained from tracking the specific animal and this can be achieved by limiting the animal’s activity to ensure that it remains in an appropriate location without other animals in its vicinity. This idea has been applied to monitor a pig’s weight [12] and back fat levels [13] and to monitor a laying hen’s activities [14].

With respect to motion tracking, Computer Vision Technology was first used in 1997 to track animal behavior [15]. In 1998, Sergean et al. [16] developed a tracking system using color information and segmented individual birds using contour information. Currently, Ellipse Fitting is the most common approach used to track laying hens. Fujii et al. [17] used a method based on particle filters for tracking multiple hens. However, the particle filters lost track of the hens when sudden quick movements were made. The method which was proposed by Kashiha [18] had a superior performance for tracking individual laying hens in an image area but was unable to identify and track an individual laying hen in a flock. To solve this problems, Nakarmi et al. [19] installed a RFID (Radio Frequency Identification) antenna array at the bottom of a cage and attached RFIDs to the feet of hens’ to determine their location for further tracking in the distance image. Although this method can achieve suitable tracking results, it is very limited in its application. It is not conducive to practical application and wearing the RFID can lead to discomfort for the hens which in turn may alter their behavior.

To address the challenges discussed above, a new laying-hen tracking algorithm, based on the Hybrid Support Vector Machine (HSVM) model has been proposed as a method to track a single hen within a flock raised under a floor system in real time with high robustness. The objective of this experiment was to compare the ability of this method to track individual laying hens in a flock with 5 other commonly used algorithms.

## Methods

### Experimental pen design and setup

This study was approved by the Animal Care and Use Committee of China Agricultural University (Beijing, China). As tracking targets, six 20-week-old Hyline Brown laying hens weighing an average of 1.4 kg were selected for study. The hens were allowed a 2 wk acclimation period before commencing data collection.

**Fig. 1:**
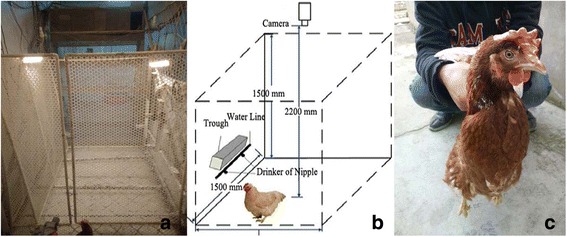
A schematic drawing and photograph of the experimental pen and observation objects. (**a**) Photograph (**b**) Schematic (**c**) Observation objects

A 1.2 m × 1.5 m pen (Fig. 1a, b) was constructed to house the birds (Fig. 1c). On two sides of the pen, LED lighting was used to illuminate the test area from 0500 h to 2100 h every day to ensure that the intensity of illumination in the pen region was approximately 15–20 lux. The hens were fed twice a day at 0900 h and 1700 h and their eggs were collected at 1700 h every day. Manure was removed daily and the barn temperature was maintained about 20 °C.

The height of the cameras used to collect video (Launch, LC5505E7-C83R) was set at 2.2 m. Videos were operated from 0500 h to 2100 h. Over 500 h of video were obtained during the subsequent 30 d. Ten 3-min fragments out of the 500 h of video were randomly chosen to validate the tracking algorithm and 778 images in the video fragments were randomly chosen and manually labeled.

### Initialization

The tracking algorithm consisted of three steps including initialization, tracking and updating. For initialization, the contour area of the target was manually marked and the rotation method was used to obtain the size of the minimum outer rectangle of the contour area. This minimum outer rectangle was represented as T_0_{w_0_,h_0_,a_0_,c_0_}, where w_0_ corresponded to the width of T_0_, h_0_ represented the height of T_0_, a_0_ was the angle between T_0_ and the x-axis, and c_0_ was the center of T_0_. This rectangle was the initial tracking rectangle and the width and height of each sample was consistent with it.

### Binary HSVM model (HSVM_b_)

The HSVM model consisted of a one-class model, a binary classification model and a regression model. Around the initial tracking rectangle, the three types of HSVM were sampled as follows. Firstly, the Binary Classification Support Vector Machine (HSVM_b_) model was established [20]. The binary model is often used for the tracking-by-detection strategy [21, 22] used in object tracking. However, this method results in a fuzzy boundary between positive and negative samples. To handle this problem, the regression model aids in locating the target more accurately to avoid drift.

**Fig. 2:**
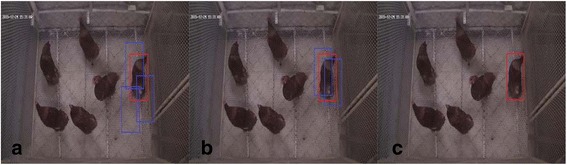
Example of sampling by the three types of support vector machines. (**a**) SVM_b_ (**b**) SVM_r_ (**c**) SVM_o_

For the HSVMb, the positive and negative samples were expressed as {x_i_, y_i_}, where y_i_ ∈{+1,0} was the label of sample x_i_. If y_i_ = 1, x_i_ was a positive sample, and x_0_ denoted the sample in the initial tracking rectangle. l(x_i_) denoted the location of sample x_i_, and l(x_0_) denoted the location of T_0_. The distance-based rule was used to select training samples [21, 23]. If ||l(x_i_)-l(x_0_)|| < d_1_, y_i_ = 1, and if d_2_ < ||l(x_i_)-l(x_0_)|| < d_3_, y_i_ = 0, (Fig. 2a) where

1 $$ {d}_2=\raisebox{1ex}{$\sqrt{W^2+{H}^2}$}\!\left/ \!\raisebox{-1ex}{$2$}\right.,{d}_1=\sqrt{20},\kern0.5em {d}_3=2\sqrt{W^2+{H}^2}\left(W={w}_0,\ H={h}_0\right) $$

To extract the histogram of orientation gradients, 50 positive and 50 negative samples were randomly selected according to the above rules. In the HSVM, the window size for the histogram of orientation gradient was 16 × 16 pixels and the cell size was 4 × 4 pixels. One block consisted of 4 cells and strided each cell once with 9 orientations. All of the samples selected for feature extraction were normalized to the size of the window. With the features and training pairs {x_i_, y_i_}, the binary HSVM model was obtained. The confidence score of a new candidate sample x_i_ was calculated by:

2 $$ con{f}_b(x)={\displaystyle \sum_i{a}_i{y}_i{k}_b\left({x}_i,x\right)} $$

where a_i_ was the Lagrange Multiplier and k_b_(x_i_, x) was the Kernel Trick [24].

### Regression SVM model (HSVM_r_)

For HSVM_r_, all of the samples satisfying d_1_ < ||l(x_i_)-l(x_0_)|| < d_2_ were selected as training samples (Fig. 2b). The bounding box overlap area ratio was chosen to generate the regression function value y_i_ of sample x_i_, which has been widely used to evaluate the accuracy of object detection [23]:

3 $$ {y}_i=\frac{area\left({x}_o\cap {x}_i\right)}{area\left({x}_o\cup {x}_i\right)} $$

where x_0_ denoted the initial tracking rectangle. Following this principle, 50 training samples were randomly selected to obtain the regression HSVM model. For any candidate in region x, its confidence score conf_r_(x) was calculated as follows:

4 $$ con{f}_r(x)={\displaystyle \sum_i\left({a}_i-{a}_i^{*}\right){k}_r}\left({x}_i^T,x\right) $$

where a_i_ and a_i_^*^ were the Lagrange Multipliers and k_r_(x_i_^T^, x) was the Kernel Trick [24].

### One-class support vector machine (HSVM_o_)

The one-class HSVM was the third model. The one-class model can be considered as an appearance model and can distinguish between individual layers [24]. Consequently, during the tracking stage, the confidence score of the candidate samples, chosen according to the tracking strategy used, was calculated using the HSVM model after feature extraction. The candidate region corresponding to the highest score was the tracking result of the current frame (Fig. 2c). After obtaining the tracking result for the current frame, we decided whether or not it was necessary to re-sample for model re-training in order to adapt to changes in target appearance.

One difference between the HSVM_o_ and the first two models was that it used the entire tracking result region of each previous frame as the training sample. The confidence score of a candidate sample x_i_ was calculated as follows:

5 $$ con{f}_o(x)={\displaystyle \sum_i{a}_i}{k}_o\left({x}_i,x\right) $$

where a_i_ was the Lagrange Multiplier and k_o_(x_i_, x) was the Kernel Trick [24].

After obtaining these three sub-models, the confidence score of a candidate sample x_i_ was calculated by

6 $$ conf(x)=\raisebox{1ex}{$\left({w}_o\ast conf{n}_o(x)+{w}_r\ast conf{n}_r(x)+{w}_b\ast conf{n}_b(x)\right)$}\!\left/ \!\raisebox{-1ex}{${w}_o+{w}_r+{w}_b$}\right. $$

where confn_o_(x), confn_r_(x), and confn_b_(x) were the results after normalizing conf_o_(x), conf_r_(x), and conf_b_(x) into the range [0,1]. w_o_, w_r_, and w_b_, corresponded to the weights of each sub-model, respectively. The weights of each sub-model determined the relative contribution of each HSVM. HSVMb, adopted the binary classification, and was robust to changes in bird pose and therefore it worked the best for monitoring preening and flapping of wings for example. HSVMr effectively solved the drift problem. It had the best results for when the test hens were close to each other. HSVMo was not sensitive to a fast-changing background and therefore had good performance to monitor sudden movements from the hens [24]. Considering the adaptation of the different support vector machines to different scenarios and the results of repeated attempts, w_o_, w_r_, and w_b_ were set to 0.3, 0.6, and 0.1.

### Tracking

In the tracking phase, the candidate samples were obtained around the tracking object. The model scoring was applied to select the best tracking results. The specific process was as follows:

- The tracking result of the previous frame was set as the initial target area T_o_{w_o,_ h_o_, a_o_, and c_o_} of the current frame.
- c_o_ was set as the center of rotation. The target area was rotated h times in clockwise and counterclockwise directions, respectively. Each rotation was deflected by k degrees. If the coordinate of point X was (x,y) before the rotation, it became (x’,y’) after the rotation and the mapping formula was
  7 $$ y\hbox{'}=\left(x-{x}_0\right)\times sin\left({a}_0+{\left(-1\right)}^ih\times k\right)+\left(y-{y}_0\right)\times cos\left({a}_0+{\left(-1\right)}^ih\times k\right)+{y}_0 $$
  8 $$ x\hbox{'}=\left(x-{x}_0\right)\times cos\left({a}_0+{\left(-1\right)}^ih\times k\right)-\left(y-{y}_0\right)\times sin\left({a}_0+{\left(-1\right)}^ih\times k\right)+{x}_0 $$
  where the coordinate of c_0_ was (x_0_,y_0_). If the rotation direction was clockwise, i = 1; otherwise i =2.
  There were a total of 2×h + 1 candidate regions. After the features were extracted from these regions, the HSVM model was used to calculate their confidence score. The candidate region with the highest score was chosen as the best tracking region T_a_{w_a_,h_a_,a_a_,c_a_}, with respect to the angle. In the current experiment, h was set to 5 and k was set to 3;
- T_a_ was expanded m times to obtain the shift search area T_m_{w_m_,h_m_,a_m_,c_m_}, where w_m_ = m×w_a_,h_m_ = m×h_a_,a_m_ = a_a_,and c_m_ = c_a_. The search box T_s_{w_s_, h_s_, a_s_, c_s_} was used to search the entire shift search area, where the initial value of the search box was w_s_ = w_a_, h_s_ = h_a_, and a_s_ = a_a_. If the coordinate of c_a_ was (x_a_,y_a_) and the coordinate of c_s_ was (x_s_,y_s_), then
  9 $$ {x}_s=-0.1\times W\times cos{\alpha}_a+0.1\times H\times sin{\alpha}_a+{x}_a $$
  10 $$ {y}_s=-0.1\times W\times sin{\alpha}_a-0.1\times H\times cos{\alpha}_a+{y}_a $$

The search box maintained the same size and angle during the search process, while displacing it by M and N steps in the indicated direction along the width and height of the search area, respectively. When the search box was moved i times along the width and j times along the height, w_s_, h_s_, and a_s_ remained unchanged, and the coordinates of c_s_ were calculated as follows:

11 $$ x\hbox{'}=\left(-\left(m-1\right)\times \frac{1}{2}\times {W}_a+\raisebox{1ex}{$\left(m-1\right)$}\!\left/ \!\raisebox{-1ex}{$M\times {W}_a\times i$}\right.\right)\times \cos {a}_a-\left(-\left(m-1\right)\times \frac{1}{2}\times {H}_a+\raisebox{1ex}{$\left(m-1\right)$}\!\left/ \!\raisebox{-1ex}{$N\times {H}_a\times j$}\right.\right)\times \sin {a}_a+{x}_a $$

12 $$ y\hbox{'}=\left(-\left(m-1\right)\times \frac{1}{2}\times {W}_a+\raisebox{1ex}{$\left(m-1\right)$}\!\left/ \!\raisebox{-1ex}{$M\times {W}_a\times i$}\right.\right)\times \sin {a}_a+\left(-\left(m-1\right)\times \frac{1}{2}\times {H}_a+\raisebox{1ex}{$\left(m-1\right)$}\!\left/ \!\raisebox{-1ex}{$N\times {H}_a\times j$}\right.\right)\times \cos {a}_a+{y}_a $$

Thus, there were a total of M×N regions. After extracting the features of these regions and scoring them using the HSVM model, the candidate region with the highest score was selected as the best region, with respect to displacement (which was an initial target region of tracking). In this study, m = 1.2, M = 5, and N = 5.

**Fig. 3:**
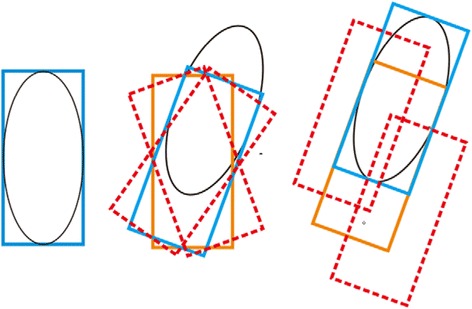
Schematic diagram of the tracking process. The tracking object is indicated by an ellipse; the blue box represents the best tracking area of the current step; the orange box represents the location of the tracking box in previous steps; the red dashed boxes represent the candidate regions. The best region is selected from the candidate regions

The steps (b) and (c) were alternated until the two adjacent quasi-tracking areas coincided. At this time, the corresponding tracking box became the tracking area of this frame image (Fig. 3).

Because histogram of orientation gradient feature extraction is relatively time-consuming, the displacement and angle of laying hens were tracked separately. Firstly, the algorithm tracked the change in the angle and subsequently the change in the displacement, and was iterated until there was no more movement. In this way, the number of sampling iterations was effectively reduced. This method had no significant impact on the final results and effectively improved the real-time performance of the algorithm. For instance, in an iterative process, the number of sampling iterations of the tracking strategy was M×N + 2H + 1, while this number increased to (M×N)×(2H + 1) if the displacement and angle were tracked simultaneously.

### Updating

Because a hen uses a non-rigid body motion, its appearance may change significantly during movement, especially if it turns, or if some of its body is partially obscured. To accommodate the hens’ changing appearance during movement, the model must be updated.

The degree of change in appearance had to be calculated after the end of each frame of video tracking to determine if it required updating [24]:

13 $$ d\left({x}_{cur},{x}_j\right)=1- \max \frac{\left\langle {x}_{cur},{x}_j\right\rangle }{\left\Vert {x}_{cur}\right\Vert \bullet \left\Vert {x}_j\right\Vert } $$

In the above formula, x_cur_ was the characteristic value of the tracking result of the current frame and x_j_ was the characteristic value of the previous tracking results of each frame.

**Fig. 4:**
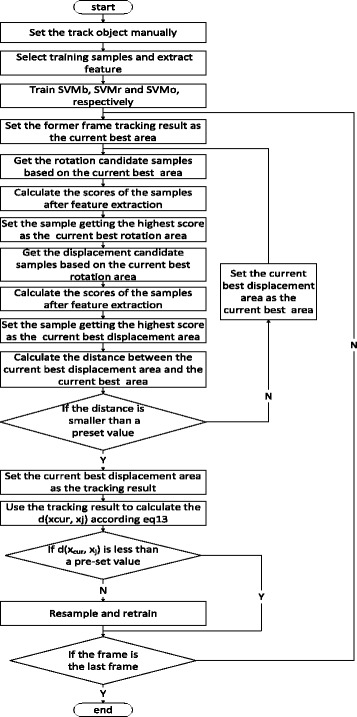
The flow chart of the HSVM tracker

If d(x_cur_, x_j_) was less than a pre-set value (0.05 in our experiment), the data was re-sampled and then retrained for the model. The re-sampling rules were as follows:

- For the binary HSVM, the image area corresponding to the image tracking box was taken as a positive sample to be inserted into the queue of 40 positive samples using the first-in-first-out strategy. Sometimes the target was blocked for a considerable duration of time and all of the positive samples corresponded to the blocked target. This could have resulted in drift problems. This problem was solved by reserving the 10 initial positive samples. In addition, 50 negative samples were randomly selected to replace all of the former negative samples.
- For the regression HSVM, the sampling method for positive samples was the same as the method for the binary HSVM. For negative samples, 20 negative samples were randomly selected to replace the original negative samples.
- For the one-class HSVM, the sampling method for positive samples was the same as the method for the binary HSVM. The whole algorithm process is shown in (Fig. 4).

## Results and discussion

The two most important criteria for the evaluation of algorithm tracking methods are real-time operation and robustness. The HSVM was implemented in OpenCV on a personal computer with a 3.50GHz Intel® Core™ i2-4150. It achieved an average speed of about 9.1 frames per second.

**Fig. 5:**
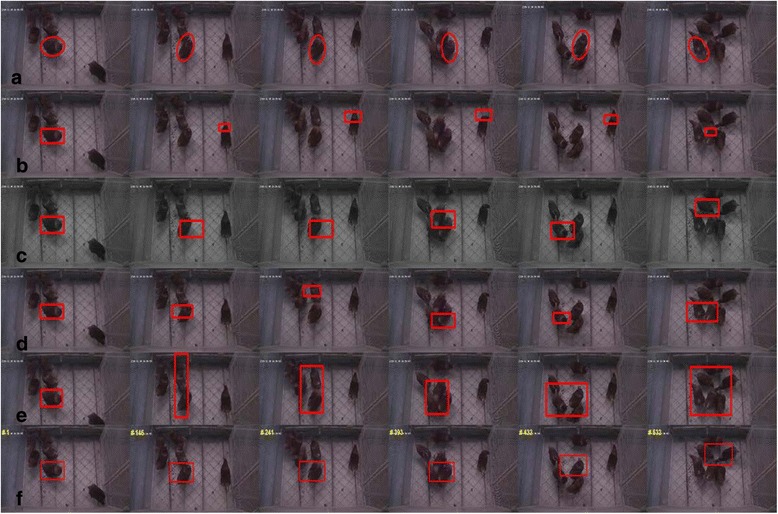
Experimental results of the six algorithms: (**a**) HSVM; (**b**) TLD; (**c**) Frag; (**d**) Particle filter; (**e**) MeanShift; (**f**) PLS

One Hyline Brown hen was chosen from the 6 observation objects as the tracking target. HSVM was compared with 5 other algorithms including Frag [25], TLD [26], PLS [27] (these three algorithms can all be downloaded from the homepage of the original author), MeanShift, and the Particle Filter Algorithm (these two are widely used classical algorithms). Each of these algorithms were used to track the target hen in the experimental video. Three experiments with 3 different randomly-selected tracking targets were conducted and the 6 algorithms were compared in these 3 experiments. The results are shown in (Fig. 5).

To assess the robustness of the algorithm, the overlap rate (OR) was used to quantify the tracking accuracy. The overlap rate was calculated as:

14 $$ OR=\frac{area\left({R}_t{\displaystyle \cap {R}_l}\right)}{area\left({R}_t{\displaystyle \cup {R}_l}\right)} $$

where R_t_ represented the results of the tracking and R_l_ represented the ground truth.

**Fig. 6:**
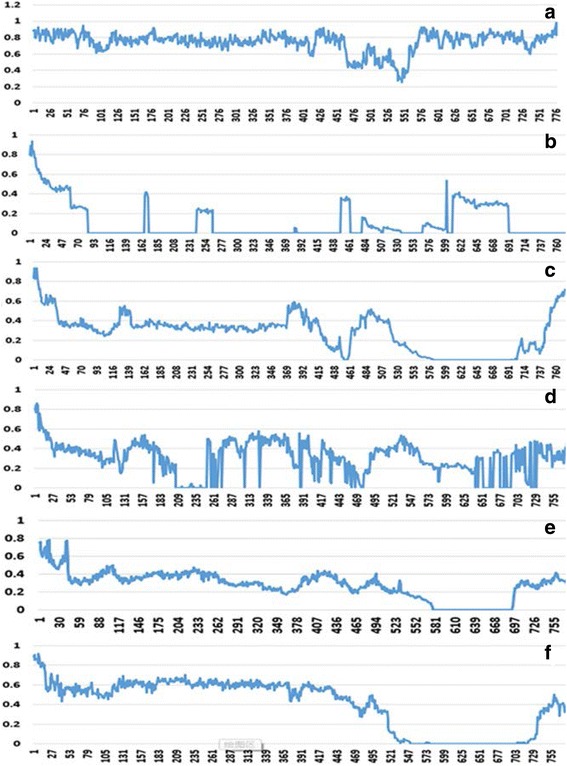
Overlap rate of the six algorithms: (**a**) HSVM; (**b**) TLD; (**c**) Frag; (**d**) Particle filter; (**e**) MeanShift; (**f**) PLS. The vertical axis of the statistical graph represents the overlap rate. Higher overlap rate scores indicate more accurate tracking, while an overlap rate of 0 indicates that the algorithm completely lost the tracking targets. The horizontal axis of the statistical graph represents the frame number of the images which have been labeled

The overlap rates were calculated for the 6 aforementioned algorithms (Fig. 6). The vertical axis of the statistical graph represented the overlap rate. Higher overlap scores indicated more accurate tracking while an overlap rate of 0 indicated that the algorithm completely lost the tracking targets. Figure 6a shows that for most frames, HSVM maintained an overlap rate of approximately 0.8.

An aggregation of the laying hens occurred during the 430th–600th frames. The hens’ mutual occlusion sent the overlap rate on a downward trend but the algorithm self-adjusted to recover an overlap rate of approximately 0.8. Figure 6b shows the statistical graph for the TLD algorithm.

The overlap rate curve dropped significantly at the beginning, indicating that the drift of the tracking box increased until the tracking box missed the target. The tracking box only rebounded to the target for a short period of time in the middle part of the frames. Figure 6c shows the graph for the Frag algorithm. The overlap rate curve decreased until the overlap rate was approximately 0.4 because the target hen kept changing its direction of movement. The curve then maintained this value for some time. After the 430th frame, the overlap rate curve declined again until the tracking box missed the target because of the aggregation of hens.

The Particle Filter Algorithm lost and retrieved the target frequently during the tracking process. As a result, the value of its overlap rate varied between 0 and 0.5, as shown in Fig. 6d, but it quickly recovered the target hen each time it lost it. Figure 6e shows that the MeanShift Algorithm tracking boxes expanded easily when the target hen got close to other laying hens resulting in the decline of the overlap rate curve. When the hens aggregated around the 430th frame, the tracking box simply expanded instead of losing the target. Therefore, after the 430th frame, the overlap rate curve did not suffer an obvious drop. The tracking box lost the target and stayed on the flock of hens when the target hen left the flock. Subsequently, the tracking box was transferred to other laying hens until the target hen and tracking box coincided again. The overlap rate curve of the PLS algorithm showed relatively stable performance, overall, and the value of overlap rate was approximately 0.6. Even so, the curve began to decline around the 430th frame until the tracking box lost the target.

From the figures described above, each algorithm adapted to different situations in the movement of laying hens. The average overlap rate is shown in Table 1 according to the different scenarios in the 778 images.

**Table 1:** Average overlap rate for the six algorithms conducted for different scenarios

Average overlap rate	HSVM	TLD	Frag	Particle Filter	MeanShift	PLS
Change of direction	0.79	0.23	0.36	0.36	0.40	0.55
Two hens mutual occlusion	0.78	0.03	0.39	0.37	0.37	0.61
Preening	0.75	0.05	0.33	0.21	0.36	0.62
Multi hens mutual occlusion	0.68	0.09	0.17	0.27	0.18	0.23
All frames	0.75	0.11	0.28	0.30	0.28	0.40

Table 1 shows that HSVM obtained a higher average overlap rate than the other algorithms both with respect to the total average overlap rate and for the different particular scenarios. The value of the overall average overlap rate was 35 % higher than the highest value among the other algorithms. When tracking a single target in a multi-hen mutual occlusion situation (the most challenging scenario), HSVM’s average overlap rate was 68 %, which was 41 % higher than the highest value attained for the other algorithms. HSVM was relatively stable with the average overlap rate maintained between 68 and 79 % across the specific cases and the overall average. The PLS algorithm attained the best performance among the contrast algorithms because the PLS was able to model the correlation of target appearance and class labels due to its capacity for both dimensionality reduction and classification [27]. The value of the average overlap rate for the changing of direction, two hens’ mutual occlusion, and preening scenarios was 55, 61 and 62 % respectively. However, PLS performed poorly in handling the heavy occlusion, which can easily and quickly change the appearance of targets [28]. In the situation of multiple hens’ mutual occlusion, PLS lost the target hen for some frames resulting in a drop in the average overlap rate to 23 %. For the situation of multiple hens’ mutual occlusion, the best performance (excluding that of HSVM) was achieved by the Particle Filter Algorithm, whereby the average overlap rate only reached 27 %.

The TLD algorithm used the optical flow method to track the object, which meant the following three conditions had to be satisfied. First, the change of luminance in the different frames should be very small. Secondly, the content of two adjacent frames should change very slowly. Finally, the projections of nearby image points were nearby points and shared similar speed [29]. The lighting in our hen house was not uniform and could not be kept stable. Moreover, hens often made sudden and quick movements such that the average overlap rate of TLD was only 11 %. The reason is that the true target was blurred, and it was difficult for the TLD to distinguish it from the background [30].

The MeanShift tracker had the advantage of low complexity, but it also failed with fast motion, illumination changes, cluttered background and occlusion [31, 32]. The average overlap rate of the MeanShift tracker was only 17 % higher than that of TLD. The Particle Filter Algorithm tracked the object by predicting its location in the next frame. It worked well when the object was briefly blocked. However, if the occlusions lasted for a longer duration, the tracking was more likely to fail [33]. Furthermore, the Particle Filter Algorithm lost the target during quick or sudden movements [17]. Thus, the average overlap rate of the Particle Filter Algorithm was similar to that of the MeanShift tracker. The Frag can cope with many different situations due to the use of local appearance models [34]. But Frag performed poorly in this experiment because it could not handle drastic appearance changes [35–37], so the average overlap rate of Frag was only 28 %.

Figure 6 and Table 1 demonstrate that our HSVM tracker was superior to the classical methods and existing state-of-the-art methods, with respect to better coverage and robustness on the testing sequences.

HSVM owes its success to the following aspects. First, the algorithm used histogram of orientation gradient features to detect laying hens and this effectively described the contour of the laying hens. Secondly, a new type of tracking strategy that accounted for the laying hens’ displacement and their body angle improved the tracking accuracy. Third, although the histogram of orientation gradient feature extraction was time-consuming, the algorithm still had a good real-time performance by optimizing the tracking process and reducing the number of sampling iterations.

**Fig. 7:**
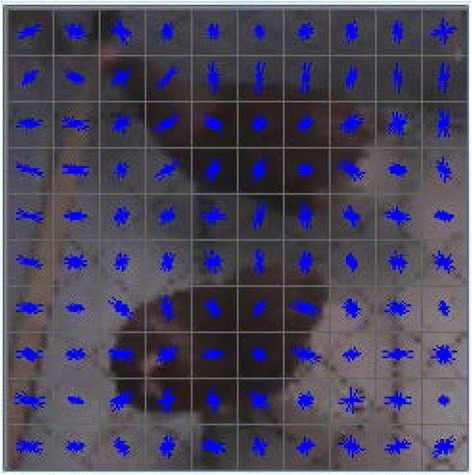
Visualization results of the histogram of orientation gradient feature used in the HSVM track

Although the HSVM algorithm showed impressive potential, there are still areas that need improvement. The histogram of orientation gradient feature was based on the object edge gradient (Fig. 7). Thus, if the tracking object is significantly occluded for a long time, the HSVM algorithm may also lose track of the object. In this experiment, the stocking density was not too high, and this situation happened only a few times in the videos. In further research, the stocking density will be increased to explore approaches to improve the robustness of the algorithm.

## Conclusions

In this paper, a laying hen tracking algorithm based on the HSVM was developed to track a single hen within a flock of hens under a floor system. The experimental results showed that the algorithm achieved better robustness and real-time performance than other comparable algorithms, indicating that HSVM has a substantial practical value in the field. Because it does not require the support of a sensor, the HSVM had better application prospects. With the tracking approach, we can classify the laying hens’ behavior to achieve automatic recognition. To improve the average overlap rate in future work, we will investigate a method to adjust the size of the tracking box based on the size change of the moving tracking targets.
